# Gastrointestinal symptoms in patients with postural orthostatic tachycardia syndrome in relation to hemodynamic findings and immunological factors

**DOI:** 10.3389/fphys.2024.1342351

**Published:** 2024-01-29

**Authors:** Hanna Tufvesson, Viktor Hamrefors, Artur Fedorowski, Monika Hansson, Bodil Ohlsson

**Affiliations:** ^1^ Department of Clinical Sciences, Lund University, Malmö, Sweden; ^2^ Department of Gastroenterology, Skåne University Hospital, Malmö, Sweden; ^3^ Department of Cardiology, Skåne University Hospital, Malmö, Sweden; ^4^ Department of Medicine, Karolinska Institute, and Department of Cardiology, Karolinska University Hospital, Stockholm, Sweden; ^5^ Department of Medicine, Division of Rheumatology, Karolinska Institute, and Karolinska University Hospital, Stockholm, Sweden; ^6^ Department of Internal Medicine, Skåne University Hospital, Malmö, Sweden

**Keywords:** postural orthostatic tachycardia (POTS), gastrointestinal tract, tilt table test, autoimmunity, ehlers-danlos syndrome, irritable bowel syndrome, mast cells

## Abstract

Gastrointestinal (GI) symptoms are common in postural orthostatic tachycardia syndrome (POTS). We aimed to explore the prevalence and severity of GI symptoms in POTS, and to investigate immunological factors, hemodynamic findings, and their possible association with GI symptoms in POTS. Forty-three patients (93% female, median age 30.6 (26.0–41.0) years), previously diagnosed with POTS and 74 healthy controls (78% female, median age 35.6 (28.8–41.7) years) were included. The participants completed a questionnaire including prevalence of GI symptoms, the irritable bowel syndrome severity scoring system (IBS-SSS), and visual analog scale for IBS (VAS-IBS). All POTS patients were previously examined by tilt test (2010–2021) and the vast majority with more recent active standing test (2017–2021), which included monitoring of heart rate (HR). ΔHR was calculated as difference between supine and upright position. Continuous variables from IBS-SSS and VAS-IBS were correlated to ΔHR. A microarray containing several autoantigens commonly targeted in systemic autoimmune disorders was used to assess prevalent autoantibodies in POTS and controls. Total IgE and S-tryptase were analyzed. GI symptoms were more prevalent and severe in POTS than in controls; nausea being the most prevalent (79.1% vs 4.9%, *p* < 0.001) and bloating and flatulence being the most severe (median 65 (25–88) vs 0 (0–14), *p* < 0.001). The median total IBS-SSS was 213 (135–319) in POTS vs 13 (0–54) in controls (*p* < 0.001). Total IBS-SSS was associated with low psychological wellbeing (r = 0.539, *p* < 0.001) in POTS. ΔHR_max_ correlated inversely with abdominal pain (r = −0.406, *p* = 0.007). After adjustments for psychological wellbeing, total IBS-SSS still associated inversely with ΔHR_10min_ (β: 4.748; 95% CI: −9.172 to −0.324; *p* = 0.036). Similar results were seen with active standing test. The prevalence of autoantibodies did not differ between POTS and controls (29.4% vs 33.3%, *p* = 0.803). There was no association between GI symptoms and autoantibody status. Total IgE and tryptase were elevated in a few cases. This study confirms the high prevalence of GI symptoms in POTS. More pronounced tachycardia upon tilt table testing seems to be inversely correlated with severity of chronic GI symptoms in POTS. This study did not support the hypothesis that POTS is associated with immunological factors.

## 1 Introduction

Postural orthostatic tachycardia syndrome (POTS) is a cardiovascular autonomic disorder, characterized by a heart rate (HR) increase >30 beats per minute (bpm) or a HR of >120 bpm within 10 min upon standing, and no significant reduction in blood pressure (BP) (Brignole et al., 2018; Vernino et al., 2021). To fulfill the diagnostic criteria, the patient must have chronic symptoms (>3 months) of orthostatic intolerance, i.e., palpitations, blurred vision, fatigue, and lightheadedness upon standing that improve when the patient returns to supine position. Other causes of tachycardia and orthostatic intolerance, such as anemia, should be ruled out (Vernino et al., 2021; Raj et al., 2022). As diagnostic tool, the head up tilt test can be performed to measure HR and BP responses from supine to standing (Brignole et al., 2018). The more easily performed active standing test can be used as well, with the same diagnostic criteria (Brignole et al., 2018; Fedorowski, 2019). The epidemiology of POTS is not fully explored, but previous studies estimate a prevalence ranging from 0.2% to 1.0% in the U.S. population, with females over-represented with a 4:1 ratio, especially in younger ages (Fedorowski, 2019). POTS frequently presents with chronic comorbidities or conditions that are unrelated to orthostatic intolerance, such as migraine, fibromyalgia, chronic fatigue, and allergic disorders (Liao et al., 2017; Chelimsky and Chelimsky, 2018). Hypermobile spectrum disorders (HSD) including hypermobile Ehlers-Danlos syndrome (hEDS) are also more common in POTS (Miller et al., 2020; Tai et al., 2020). Autoimmunity has been proposed as a possible etiological mechanism in POTS, with high prevalence of diverse antibodies in previous reports (Fedorowski, 2019). Moreover, increased prevalence of co-morbid systemic autoimmune rheumatic disorders have been reported in POTS (Mannan and Pain, 2023), but causative autoantibodies have not been found so far (Vernino and Stiles, 2018). Mast cell activation disorder has been proposed to be associated with POTS with atypical symptom presentation, such as predominant gastrointestinal (GI) symptomatology (Kohno et al., 2021).

GI symptoms are very frequent in POTS, well described in several studies (Chelimsky and Chelimsky, 2018; DiBaise et al., 2018; Mehr et al., 2018; Tu et al., 2020). Some GI symptoms may be triggered by orthostatic challenge, and are thereby relieved in recumbent position, whereas most other GI complaints are non-orthostatic (Chelimsky and Chelimsky, 2018; Tu et al., 2020). The enteric nervous system is a part of the autonomous nervous system (Gibbons, 2019), receiving input from parasympathetic and sympathetic nerves, as well as input from the central nervous system (Koloski et al., 2012; Mehr et al., 2018). Disturbances of the enteric nervous system can cause GI disorders such as gastroparesis, and symptoms like pain and altered bowel habits (Camilleri, 2021). Since POTS is associated with disturbances of the cardiovascular autonomic nervous system (Vernino et al., 2021), our hypothesis was that more exaggerated pulse increase on tilt testing is associated with more severe GI symptoms, as a surrogate marker of autonomic and enteric nervous dysfunction.

Consequently, the first aim of this study was to explore GI symptoms in a POTS cohort compared with healthy controls using validated GI symptom questionnaires, and to investigate whether GI symptoms correlated with pulse increase during tilt test as a surrogate of disease severity. The second aim was to explore the prevalence of common autoantibodies found in systemic autoimmune rheumatic disorders, as well as markers of allergy and mast cell activation in POTS, compared with controls, and to investigate whether these immunological factors are associated with GI symptoms.

## 2 Materials and methods

### 2.1 Study design

Study participants were recruited at the Department of Gastroenterology, Skåne University Hospital, Malmö, Sweden between October 2020 and January 2022. Invitations including study information were sent by regular mail to patients with previously confirmed POTS by tilt testing during 2010–2021 at the Department of Cardiology at the same hospital. One of the authors (HT) contacted the patients by telephone, to give further information and book a visit. During the same time frame, healthy controls were recruited among hospital staff and students. The study participants were asked to fill in study questionnaires during the study visit where a clinical examination and blood sampling were performed (Tufvesson et al., 2023). All blood samples were drawn in a non-fasting state, and the patients took their regular medications prior to the study visit.

### 2.2 Study population

#### 2.2.1 The POTS cohort

##### 2.2.1.1 Patients

The Syncope Study of Unselected Population in Malmö (SYSTEMA) cohort, with over 3,000 patients investigated for syncope and severe orthostatic intolerance at the Skåne University Hospital in Malmö, Sweden, was conducted between 2008 and 2021. Details of the SYSTEMA cohort are described elsewhere (Fedorowski et al., 2013). From the SYSTEMA cohort, 93 patients with a clinically confirmed POTS were included in a POTS sub-study cohort with additional tests, at the Clinical Research Unit, Department of Internal Medicine, during 2017–2020 (Spahic et al., 2023).

##### 2.2.1.2 Cardiovascular autonomic testing

During inclusion in the SYSTEMA cohort 2008–2021, the patients underwent cardiovascular autonomic tests with tilt testing and continuous hemodynamic monitoring (Johansson et al., 2021). Moreover, all patients had been thoroughly evaluated by a senior expert (AF) to exclude other primary causes of the hemodynamic findings. The tilt testing protocol included supine rest for 10 min preceding table elevation to 60°–70° for 20 min. Data on BP and HR derived from 30 s stable periods were collected at supine position, and after 1, 3, and 10 min upon the tilt test. Also, the maximum HR during tilt test was noted (Spahic et al., 2023). POTS diagnosis was defined as symptoms of orthostatic intolerance lasting for ≥3 months associated with a pathological tilt test showing a HR increase >30 bpm from supine position or a HR > 120 bpm in standing, with no evidence of orthostatic hypotension (Brignole et al., 2018). For each patient, a ΔHR was calculated from the supine position and the maximum HR and the HR at 1, 3 and 10 min of up-right position, to measure the magnitude of chronotropic response during the tilt test.

Since some of the tilt tests were performed up to 10 years ago, we also included more recent data from active standing tests that were performed between 2017 and 2021 in the majority of the POTS-patients that were included in the current study. ΔHR was calculated from supine position and for 1, 3, and 5 min after standing as well as maximal HR. When re-tested with active standing test, the patients were asked to discontinue cardiovascular pharmacological agents 48 h prior to examination (Spahic et al., 2023).

#### 2.2.2 Study participants

##### 2.2.2.1 Patients

Eligible patients from the POTS sub-study (*n* = 82) obtained written information about the present study if they were living within a reasonable proximity to Malmö, Sweden. If the patients were interested in participating in the study, they were asked to contact one of the authors (HT), who later contacted them by telephone to give further information. The inclusion criteria were age 18–70 years and ability to fully understand the study information.

##### 2.2.2.2 Control subjects

Healthy control participants were recruited among hospital staff and medical students at Skåne University Hospital, Malmö, through personal invitation and advertisement. The controls were not allowed to have any current chronic or acute illness or significant GI symptoms. Intake of multivitamins and hormonal contraceptive medicines was accepted, as well as temporary use of medications, such as seasonal allergy medicines and pain killers.

### 2.3 Clinical examinations

During the study visit, POTS patients were examined with heart-, lung-, abdominal-, and neurological status. The current weight and height were obtained, as well as resting BP from the right arm, in a supine position after approximately 5 min of rest. The healthy controls were examined with height, weight, resting BP, and HR.

### 2.4 Questionnaires

#### 2.4.1 Study questionnaire

All study participants were asked to complete a questionnaire regarding sociodemographic factors, lifestyle habits, pregnancies and childbirth, previous and current illnesses, family history, current pharmacological treatment, and specific lifestyle modifications in case of GI complaints.

#### 2.4.2 Gastroparesis questionnaire

The gastroparesis questionnaire has been used previously in studies regarding symptoms in diabetic gastroparesis (Faraj et al., 2007). This modified questionnaire contains 14 items common in gastroparesis, where the respondent marks whether the symptom is prevalent or not. The symptoms covered are loss of appetite, dysphagia, meal-related cough, early satiety, nausea, vomiting, weight loss, abdominal fullness, bloating, regurgitation, constipation, diarrhea with gas, evacuation incontinence, and postprandial perspiration. Symptoms that are seen specifically in diabetic gastroparesis including postprandial glycemia pitfalls and symptomatic postprandial hypoglycemia were excluded in the present study.

#### 2.4.3 The visual analog scale for irritable bowel syndrome (VAS-IBS)

The VAS-IBS is a validated questionnaire regarding GI symptoms common in IBS (Bengtsson et al., 2007). The respondent marks their degree of symptoms for the past 2 weeks on a visual analog scale (VAS) between 0 and 100 mm, where 100 mm means very severe symptoms. The items covered are abdominal pain, diarrhea, constipation, bloating and flatulence, vomiting and nausea, psychological wellbeing, and the intestinal symptoms’ influence on daily life. The scales are inverted from the original version (Bengtsson et al., 2007).

#### 2.4.4 The irritable bowel syndrome severity scoring system (IBS-SSS)

The IBS-SSS questionnaire is a validated scoring system used in IBS, consisting of four items regarding abdominal pain, abdominal distension, satisfaction with bowel habit, and the impact of bowel habits on daily life, measured in mm on a 0–100 mm VAS, where 100 mm means very severe symptoms. In addition, one question asks the number of days of abdominal pain in the last 10 days. Scores from these questions are added, producing total IBS-SSS, with a maximum score of 500 (Francis et al., 1997). Scores between 75 and 175 suggests mild IBS, 175–300 suggest moderate IBS, and >300 suggest severe IBS. Additionally, there are ten extraintestinal items (nausea, early satiety, headache, back pain, lethargy, excess wind, heartburn, urinary symptoms, thigh pain, and bodily pain) measured on a VAS, that together produce an extraintestinal score, with a maximum score of 500. More severe symptoms are associated with higher scores (Francis et al., 1997; Ohlsson, 2022). In the current study, total IBS-SSS scores and extraintestinal scores were used.

### 2.5 Laboratory analyses

#### 2.5.1 Microarray

The presence of autoantibodies was evaluated using a custom-designed multiplex solid-phase microarray platform (Thermo Fisher Scientific Immunodiagnostics, Uppsala, Sweden) containing 33 different peptide and protein autoantigens targeted in systemic autoimmune rheumatic diseases, as well as control antigens (Hansson et al., 2012). The list of antigens used in the assay can be found in Supplementary Table S1. The cut-off level for each individual antigen is based on the 98th percentile value among 400 healthy controls.

#### 2.5.2 Total IgE and tryptase

Total IgE and tryptase were analyzed in serum according to clinical routines at the Department of Clinical Chemistry, Skåne University Hospital, Malmö. Reference values for healthy volunteers are given for tryptase (Lab Medicine, 2023b) and total IgE (Lab Medicine, 2023a).

### 2.6 Statistical analyses

Comparisons between groups were performed by Mann-Whitney *U* test or Fisher’s exact test as appropriate. Spearman’s correlation test was used to assess correlations between ΔHR parameters and symptoms, and between psychological wellbeing and symptoms. Generalized linear model were performed to calculate associations after adjustment for psychological wellbeing, using continuous parameters of the VAS-IBS, total IBS-SSS, total extra-intestinal IBS-SSS and all extraintestinal symptoms of the IBS-SSS as dependent variables, and ΔHR at 1, 3, and 10 min as well as ΔHR_max_ as predictors. Sensitivity analyses were performed excluding patients with concomitant organic GI disorders. Values are presented as median and interquartile range, numbers and percentages, or *ß*-value with 95% confidence interval (CI). *p* < 0.05 was considered statistically significant. Statistical analyses were performed using software SPSS^©^, version 28 for Windows (IBM, New York, United States).

### 2.7 Ethics

The present study was performed in accordance with the Declaration of Helsinki, and it was approved by the Ethics Review Board at Uppsala University, Dnr 2020–02432 and 2021–00049. The Regional Ethical Committee in Lund, Sweden, approved the SYSTEMA project (82/2008) and the POTS sub-study (2017/295).

## 3 Results

### 3.1 Clinical characteristics

In total, 43 of 82 contacted patients with POTS and 74 healthy control subjects were recruited to the present study. The median age was 30.6 (26.0–41.0) years in POTS and 35.6 (28.8–41.7) years in controls (*p* = 0.068), and the median body mass index was 24.2 (21.3–27.0) kg/m^2^ in POTS and 22.8 (21.4–25.3) kg/m^2^ in controls (*p* = 0.413). A vast majority, 93.0%, were female in the POTS group, compared with 78.4% in controls (*p* = 0.041). At clinical examination, there were no major pathological findings in the POTS group regarding heart-, lung-, and abdominal status.

The median time from definite POTS diagnosis by tilt test to the current study examination was 3.0 (1.0–7.0) years. The most common self-reported comorbidities in POTS were hypermobile spectrum disorders/Ehlers-Danlos syndrome (HSD/EDS) (*n* = 12, 28%), IBS (*n* = 12, 28%), asthma (*n* = 9, 21%), migraine (*n* = 7, 16%), and neuropsychiatric disorders (*n* = 6, 14%). Ten patients (23%) reported comorbid autoimmune disorders (psoriasis, psoriasis arthritis, lichen sclerosus, autoimmune thyroid disorders, and celiac disease), and another five patients (12%) reported other inflammatory disorders (inflammatory bowel disease, microscopic colitis, and rosacea) (Supplementary Table S2). Four of the patients who reported HSD/EDS also reported IBS. Seven patients (16%) reported organic GI disorder, including celiac disease (*n* = 2), inflammatory bowel disease (*n* = 2), microscopic colitis (*n* = 2), and persistent *helicobacter pylori* infection due to antibiotic resistance (*n* = 1). Two of the patients with organic GI disorder also reported IBS. Pharmacological treatment was abundant in the POTS group, especially cardiovascular and anti-allergy drugs. Notably histamine H1-blockers were taken by 13 patients (Supplementary Table S3). In total, 15 patients reported asthma, allergy, or were treated with anti-allergy drugs.

The tilt testing variables including HR and BP are presented in Table 1. In total, 38 (88%) patients performed an active standing test in the POTS sub-study, and variables are presented in Table 2. Nine of these patients did not manage to discontinue all of the cardiovascular medications 48 h before the examination.

**TABLE 1:** Head up tilt test-data 2011–2021.

	POTS N = 43
Systolic BP supine	127 (117–136)
Diastolic BP supine	78 (65–86)
HR supine	85 (71–91)
Systolic BP 1 min	137 (126–144)
Diastolic BP 1 min	96 (85–105)
HR 1 min	107 (98–122)
ΔHR _1 min_	28 (21–33)
Missing data	7
Systolic BP 3 min	130 (122–140)
Diastolic BP 3 min	93 (83–104)
HR 3 min	114 (102–127)
ΔHR _3 min_	29 (25–41)
Systolic BP 10 min	127 (118–140)
Diastolic BP 10 min	91 (84–103)
HR 10 min	117 (102–126)
ΔHR _10 min_	34 (28–40)
Missing data	10
Maximum HR	124 (114–139)
ΔHR _max_	44 (36–53)

Values presented as median and interquartile range. BP, blood pressure; HR, heart rate.

**TABLE 2:** Active standing test 2017–2019.

	POTS N = 38
Systolic BP supine	116 (104–123)
Diastolic BP supine	69 (65–79)
HR supine	72 (64–81)
Systolic BP 1 min	118 (109–132)
Diastolic BP 1 min	81 (77–92)
HR 1 min	99 (89–110)
Δ HR 1 min	26 (16–34)
Missing data	1
Systolic BP 3 min	116 (106–126)
Diastolic BP 3 min	80 (75–86)
HR 3 min	104 (88–111)
Δ HR 3 min	27 (23–35)
Systolic BP 5 min	116 (108–126)
Diastolic BP 5 min	79 (73–87)
HR 5 min	95 (83–114)
Δ HR 5 min	28 (18–37)
Missing data	2

Values presented as median and interquartile range. BP, blood pressure; HR, heart rate.

### 3.2 Gastrointestinal symptom scoring systems

**FIGURE 1:**
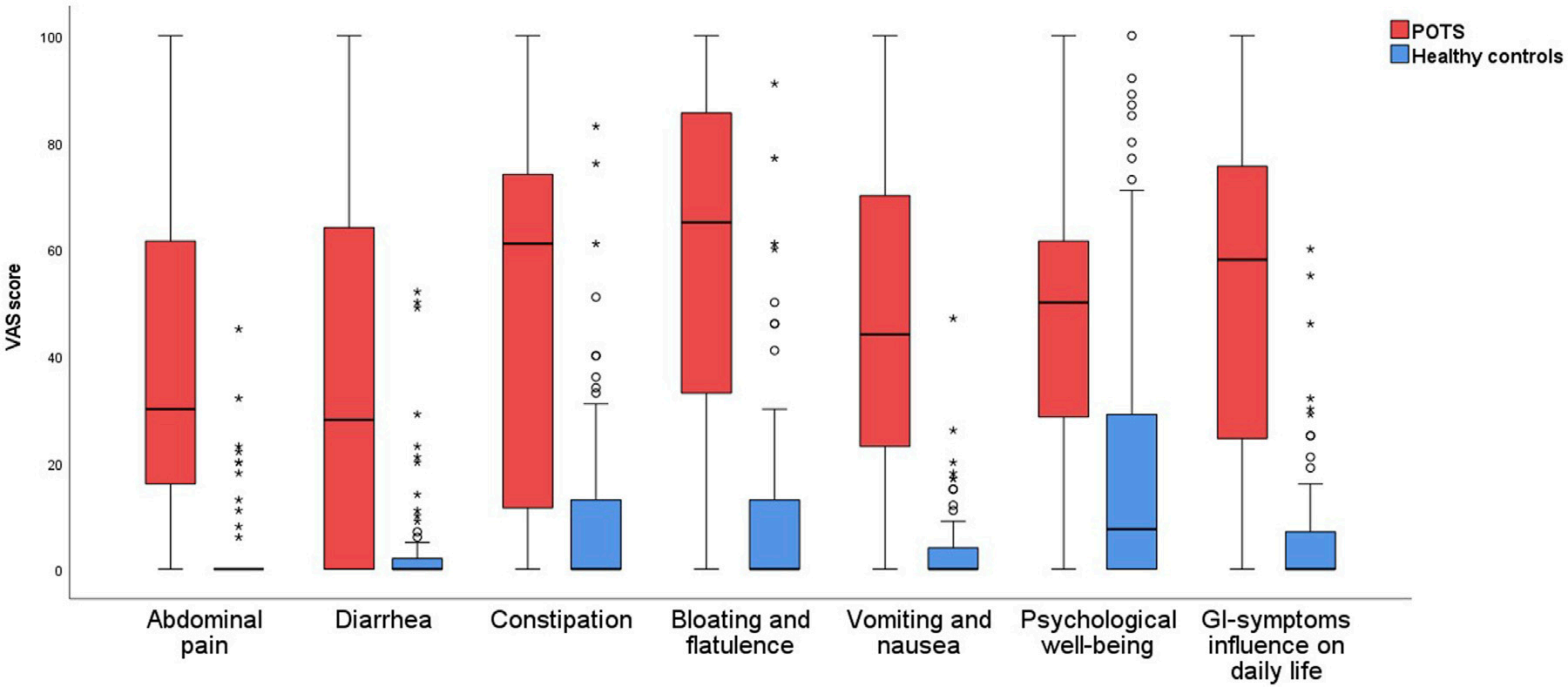
Gastrointestinal symptoms in patients with POTS and healthy control subjects, measured in millimeters on the VAS-IBS scale.

All items on the gastroparesis score were significantly more prevalent in POTS than in controls (*p* < 0.01), and both specific and overall GI symptoms were more severe in POTS than in controls (*p* < 0.001) (Table 3; Figure 1). Nausea was the most prevalent symptom reported in POTS (79.1%), followed by abdominal fullness (69.8%) and bloating (66.7%) (Table 3). The most severe symptoms were bloating and flatulence (65 (25–88)) and constipation (61 (11–74)) (Table 3; Figure 1).

**TABLE 3:** Gastrointestinal and autonomic symptoms in POTS and healthy controls.

	Healthy controls N = 74	POTS N = 43
**Gastroparesis score**		
Loss of appetite	1 (1.6%)	20 (46.5%)
Dysphagia	0 (0%)	16 (37.2%)
Meal-related cough	1 (1.6%)	14 (32.6%)
Early satiety	2 (3.3%)	23 (53.5%)
Nausea	3 (4.9%)	34 (79.1%)
Vomiting	0 (0%)	6 (14.3%)
Weight loss	0 (0%)	13 (31.0%)
Abdominal fullness	7 (11.5%)	30 (69.8%)
Bloating	4 (6.6%)	28 (66.7%)
Regurgitation	2 (3.3%)	23 (53.5%)
Constipation	10 (16.4%)	28 (65.1%)
Diarrhea with gas	5 (8.2%)	23 (53.5%)
Evacuation incontinence	1 (1.6%)	10 (23.3%)
Postprandial perspiration	0 (0%)	17 (39.5%)
**VAS-IBS**		
Abdominal pain	0 (0–0)	30 (16–62)
Diarrhea	0 (0–2)	28 (0–64)
Constipation	0 (0–13)	61 (11–74)
Bloating and flatulence	0 (0–14)	65 (25–88)
Vomiting and nausea	0 (0–4)	44 (23–70)
Psychological wellbeing	8 (0–30)	50 (28–62)
Symptoms’influence on daily life	0 (0–7)	58 (23–76)
Urgency	4 (5.6%)	17 (40.5%)
Incomplete evacuation	20 (27.4%)	30 (69.8%)
**IBS-SSS**		
Total IBS-SSS	13 (0–54)	213 (135–319)
Nausea	0 (0–4)	44 (23–70)
Early satiety	0 (0–0)	39 (5–60)
Headache	9 (0–23)	71 (33–85)
Back pain	6 (0–19)	50 (15–77)
Lethargy	16 (2–32)	92 (81–100)
Excess wind	0 (0–14)	60 (29–82)
Heart burn	0 (0–5)	23 (3–60)
Urinary symptoms	0 (0–4)	44 (8–84)
Thigh pain	0 (0–0)	42 (2–58)
Bodily pain	0 (0–12)	76 (29–96)
Total extraintestinal symptom score	29 (16–58)	277 (178–338)

Gastrointestinal symptom prevalence was assessed by the gastroparesis score. Gastrointestinal symptom severity was assessed by IBS-SSS, Irritable Bowel Syndrom-Severity Scoring System and VAS-IBS, visual analog scale for irritable bowel syndrome, 0–100 mm, where 0 represents no symptoms and 100 maximal symptoms Values presented as number (percent) or median (interquartile range). Comparative analyses were performed with Fisher’s Exact test or Mann Whitney-U, test. The *p*-value was <0.001 between patients and controls in all symptoms, except the gastroparesis score vomiting, that was *p* = 0.004. A few missing values were noted.

The total IBS-SSS score was 213 (135–319) in POTS compared with 13 (0–54) in controls (*p* < 0.001). All extra-intestinal symptoms were aggravated in the POTS group, and the total extraintestinal symptom score was 277 (178–338) in POTS compared with 29 (16–58) in controls (*p* < 0.001) (Table 3).

There was no significant difference between patients with and without self-reported HSD/EDS regarding the prevalence of GI symptoms (data not shown). Constipation (74 (43–92) vs 51 (7–72), *p* = 0.03) and bodily pain (92 (68–99) vs 67 (16–84), *p* = 0.034) were more exaggerated in HSD/EDS compared with the non-HSD/EDS group (Table 4). Abdominal pain tended to be more pronounced in HSD/EDS, but the significance level was not reached (58 (24–76) vs 30 (16–50), *p* = 0.051). The HR_max_ was higher in the non-HSD/EDS group (130 (116–147) vs 119 (103–130), *p* = 0.035), as well as the ΔHR_max_ (47 (38–54) vs. 32 (30–45), *p* = 0.005) (Table 4).

**TABLE 4:** GI symptoms and tilt-data in POTS with and without HSD/EDS.

	HSD/EDS N = 12	Non-HSD/EDS N = 31	*p*-value
**VAS-IBS**			
Abdominal pain	58 (24–76)	30 (16–50)	0.051
Diarrhea	51 (0–71)	23 (0–64)	0.603
Constipation	74 (43–92)	51 (7–72)	0.030
Bloating and flatulence	64 (32–87)	70 (15–88)	0.797
Vomiting and nausea	48 (28–70)	40 (12–73)	0.655
Psychological wellbeing	60 (37–76)	50 (22–60)	0.155
Symptoms’ influence on daily life	68 (23–84)	50 (23–74)	0.408
Urgency	5 (41.7%)	12 (40%) 1 missing	1.000
Incomplete evacuation	8 (66.7%)	22 (71%)	1.000
**IBS-SSS**			
Total IBS-SSS	297 (143–387)	193 (126–296) 3 missing	0.275
Nausea	48 (28–70)	40 (12–73)	0.655
Early satiety	51 (7–75)	25 (2–54)	0.321
Headache	65 (38–79)	77 (25–93)	0.309
Back pain	56 (36–93)	50 (8–77)	0.378
Lethargy	92 (86–99)	94 (78–100)	0.913
Excess wind	73 (40–82)	50 (27–87)	0.735
Heart burn	56 (24–71)	21 (2–54)	0.139
Urinary symptoms	52 (25–81)	44 (8–85)	0.924
Thigh pain	46 (1–54)	19 (2–60)	0.892
Bodily pain	92 (68–99)	67 (16–84)	0.034
Total extraintestinal score	322 (244–354)	248 (154–316)	0.176
**Tilt-data**			
HR supine	80 (69–97)	85 (72–90)	0.807
HR 1 min	111 (93–129)	107 (99–120)	0.697
Δ HR _1 min_	30.5 (24.5–32.3)	24.5 (19.8–33.3)	0.548
Missing data	5	2	
HR 3 min	107 (95–126)	115 (104–127)	0.310
Δ HR _3 min_	37.5 (22.8–35.3)	30 (26–41)	0.233
HR 10 min	117 (94–128)	117 (102–126)	0.906
Δ HR _10 min_	30.5 (21–41.5)	34 (30–39)	0.410
Missing data	2	8	
Max HR	119 (103–130)	130 (116–147)	0.035
Δ HR _max_	32 (30–45)	47 (38–54)	0.005

Values presented as median (interquartile range), or number (percent). Comparative analyses were performed with Mann Whitney-U, test or Fisher’s Exact test. VAS-IBS, Visual Analog Scale for Irritable Bowel Syndrome; IBS-SSS, Irritable Bowel Syndrom-Severity Scoring System. HSD/EDS, hypermobile spectrum disorder/Ehlers-Danlos syndrome. HUT, Head up tilt; SBP, systolic blood pressure; DBP, diastolic blood pressure; HR, heart rate. There were 3 missing values regarding Total-IBS-SSS, and 1 regarding urgency in the non-HSD/EDS, group.

Postprandial perspiration was more prevalent in patients with self-reported IBS compared with those without IBS (66.7% vs 29.0%, *p* = 0.037). Diarrhea was more pronounced in IBS (57 (21–80) vs 10 (0–64), *p* = 0.011), but otherwise, no significant differences were found regarding the degree of GI symptoms (Table 5). There were no significant differences between patients with and without IBS regarding tilt data (data not shown).

**TABLE 5:** GI symptoms in POTS with and without IBS.

	IBS N = 12	Non-IBS N = 31	*p*-value
**Gastroparesis score**			
Loss of appetite	7 (58.3%)	13 (41.9%)	0.497
Dysphagia	3 (25%)	13 (41.9%)	0.484
Meal-related cough	3 (25%)	11 (35.5%)	0.720
Early satiety	9 (75%)	14 (45.2%)	0.099
Nausea	11 (91.7%)	23 (74.2%)	0.405
Vomiting	2 (18.2%) 1 missing	4 (12.9%)	0.644
Weight loss	2 (18.2%) 1 missing	11 (35.5%)	0.453
Abdominal fullness	9 (75%)	21 (67.7%)	0.727
Bloating	9 (75%)	19 (63.3%) 1 missing	0.719
Regurgitation	7 (58.3%)	16 (51.6%)	0.745
Constipation	7 (58.3%)	21 (67.7%)	0.723
Diarrhea with gas	9 (75%)	14 (45.2%)	0.099
Evacuation incontinence	2 (16.7%)	8 (25.8%)	0.698
Postprandial perspiration	8 (66.7%)	9 (29%)	0.037
**VAS-IBS**			
Abdominal pain	38 (30–63)	28 (8–53)	0.162
Diarrhea	57 (21–80)	10 (0–64)	0.011
Constipation	38 (12–79)	65 (11–74)	0.714
Bloating and flatulence	81 (54–95)	62 (14–80)	0.098
Vomiting and nausea	48 (27–75)	40 (10–70)	0.465
Psychological wellbeing	57 (39–60)	47 (28–63)	0.448
Symptoms’ influence on daily life	64 (51–84)	44 (28–63)	0.129
Urgency	7 (63.6%) 1 missing	10 (32.3%)	0.086
Incomplete evacuation	9 (75%)	21 (67.7%)	0.727
**IBS-SSS**			
Total IBS-SSS	250 (188–392) 1 missing	173 (91–313) 2 missing	0.093
Nausea	48 (27–75)	40 (10–70)	0.465
Early satiety	32 (19–62)	40 (0–60)	0.497
Headache	71 (61–87)	75 (25–85)	0.694
Back pain	45 (17–80)	50 (9–77)	0.807
Lethargy	91 (88–100)	92 (78–98)	0.512
Excess wind	74 (40–82)	50 (20–83)	0.350
Heart burn	22 (10–57)	25 (2–73)	0.654
Urinary symptoms	75 (25–100)	38 (7–83)	0.071
Thigh pain	15 (2–59)	44 (2–58)	0.967
Bodily pain	81 (24–97)	76 (29–93)	0.684
Total extraintestinal score	286 (201–354)	277 (154–316)	0.481

Values presented as median (interquartile range), or number (percent). Comparative analyses were performed with Mann Whitney-U, test or Fisher’s Exact test. VAS-IBS, Visual Analog Scale for Irritable Bowel Syndrome; IBS-SSS, Irritable Bowel Syndrom-Severity Scoring System.

No significant differences were found between patients with and without organic GI disorder regarding GI symptoms and tilt data (Supplementary Table S4).

No significant differences were found between patients with and without asthma/allergy/anti-allergy drugs (*n* = 15) regarding GI symptoms and tilt data (Supplementary Table S5).

### 3.3 Correlations between psychological wellbeing and gastrointestinal symptoms in POTS

There was a significant positive correlation between lower levels of psychological wellbeing and abdominal pain (r = 0.495, *p* < 0.001), diarrhea (r = 0.403, *p* = 0.007), constipation (r = 0.332, *p* = 0.030), bloating and flatulence (r = 0.352, *p* = 0.020), vomiting and nausea (r = 0.490, *p* < 0.001), intestinal symptoms’ influence on daily life (r = 0.587, *p* < 0.001), total IBS-SSS (r = 0.539, *p* < 0.001), early satiety (r = 0.535, *p* < 0.001), back pain (r = 0.321, *p* = 0.036), excess wind (r = 0.497, *p* < 0.001), heart burn (r = 0.431, *p* = 0.004), bodily pain (r = 0.336, *p* = 0.027), and total extraintestinal symptoms (r = 0.450, *p* = 0.002).

### 3.4 Correlations between hemodynamic response and gastrointestinal symptoms in POTS

The ΔHR_max_ correlated inversely with abdominal pain (r = −0.406, *p* = 0.007). There were no significant correlations between ΔHR and extraintestinal symptoms (Table 6). When the seven patients with organic GI disorders were excluded from the analyses, inverse correlations were seen between ΔHR_10min_ and abdominal pain, constipation, and total IBS-SSS, respectively, and between ΔHR_max_ and abdominal pain, intestinal symptoms’ influence on daily life, and total IBS-SSS, respectively (Table 6).

**TABLE 6:** Spearman’s rank correlation between heart rate acceleration during tilt test and gastrointestinal/extra-intestinal symptoms in POTS.

	ΔHR_1 min_	ΔHR_3 min_	ΔHR_10 min_	ΔHR_max_
**Abdominal pain**				
r	−0.055	−0.179	−0.339 (−0.439)	−0.406 (−0.453)
*p*-value	0.752	0.250	0.054 (0.022)	0.007 (0.006)
**Diarréa**				
r	−0.108	0.091	−0.162	−0.181
*p*-value	0.529	0.561	0.368	0.245
**Constipation**				
r	0.045	0.018	−0.263 (−0.380)	−0.157
*p*-value	0.795	0.909	0.140 (0.050)	0.314
**Bloating and flatulence**				
r	−0.002	0.135	−0.189	−0.082
*p*-value	0.992	0.388	0.293	0.601
**Vomiting and nausea**				
r	0.177	0.029	0.005	−0.082
*p*-value	0.301	0.852	0.980	0.602
**Psychological wellbeing**				
r	0.155	0.040	−0.025	−0.027
*p*-value	0.368	0.797	0.892	0.864
**Symptoms’ influence on daily life**				
r	0.008	0.008	−0.189	−0.293 (−0.367)
*p*-value	0.962	0.957	0.292	0.066 (0.028)
**Total-IBS-SSS**				
r	−0.075	−0.106	−0.348 (−0.540)	−0.293 (−0.458)
*p*-value	0.669	0.515	0.051 (0.004)	0.066 (0.007)
**Nausea**				
r		0.029	0.005	−0.082
*p*-value		0.852	0.980	0.602
**Early satiety**				
r	0.232	0.064	−0.113	−0.084
*p*-value	0.172	0.658	0.530	0.593
**Headache**				
r	−0.135	−0.027	−0.259	−0.046
*p*-value	0.433	0.863	0.145	0.772
**Backpain**				
r	−0.079	−0.135	−0.258	−0.138
*p*-value	0.649	0.388	0.147	0.379
**Lethargy**				
r	−0.013	−0.066	−0.138	−0.125
*p*-value	0.939	0.676	0.443	0.423
**Excess wind**				
r	0.110	0.149	−0.054	−0.061
*p*-value	0.521	0.341	0.767	0.699
**Heart burn**				
r	0.070	0.108	−0.153	−0.177
*p*-value	0.683	0.492	0.394	0.256
**Urinary symptoms**				
r	0.006	0.055	−0.163	−0.083
*p*-value	0.973	0.726	0.366	0.597
**Thigh pain**				
r	0.062	0.030	−0.224	0.008
*p*-value	0.720	0.848	0.210	0.959
**Bodily pain**				
r	0.156	−0.039	−0.013	−0.111
*p*-value	0.363	0.805	0.942	0.479
**Total extraintestinal score**				
r	0.094	0.024	−0.200	−0.146
*p*-value	0.586	0.877	0.264	0.349

Correlation analyses between heart rate acceleration during tilt test and continuous GI, symptoms performed with Spearman’s rank correlation test. In brackets r- and *p*-value in the sensitivity analysis without the patients with organic GI-disorder (*n* = 7). HR, heart rate; IBS-SSS, Irritable Bowel Syndrom-Severity Scoring System.

After adjustment for psychological wellbeing, inverse associations were found between ΔHR_10min_ and abdominal pain (β: −1.137; 95% CI: −2.157 to −0.117; *p* = 0.030) and total IBS-SSS (β: −4.748; 95% CI: −9.172 to −0.324; *p* = 0.036). An inverse association was also seen between ΔHR_max_ and abdominal pain (β: −0.915; 95% CI: −1.478 to −0.351; *p* = 0.002). Furthermore, adjustments for psychological wellbeing did not affect the results regarding correlations between hemodynamic data and GI symptoms when the seven patients with organic GI disorders were excluded from the analyses (data not shown).

No significant correlations were observed between ΔHR from the active standing test and symptoms. After exclusion of six patients with organic GI disorder, inverse correlations between GI symptoms and ΔHR was observed, including intestinal symptoms’ influence on daily life, diarrhea, bloating and flatulence, and total IBS-SSS (Table 7).

**TABLE 7:** Spearman’s rank correlation between heart rate acceleration and gastrointestinal/extraintestinal symptoms in POTS in the active standing tests.

	ΔHR_1 min_	ΔHR_3 min_	ΔHR_5 min_
**Abdominal pain**			
r	−0.091	0.013	−0.219
*p*-value	0.591	0.936	0.200
**Diarréa**			
r	−0.210 (−0.357)	−0.070	−0.113
*p*-value	0.212 (0.049)	0.674	0.511
**Constipation**			
r	0.054	−0.068	−0.218
*p*-value	0.752	0.684	0.202
**Bloating and flatulence**			
r	−0.069	0.031	−0.236 (−0.355)
*p*-value	0.686	0.854	0.166 (0.050)
**Vomiting and nausea**			
r	−0.041	0.187	0.018
*p*-value	0.811	0.260	0.915
**Psychological wellbeing**			
R	−0.052	0.081	−0.121
*p*-value	0.758	0.627	0.481
**Symptoms’ influence on daily life**			
R	−0.017 (−0.642)	0.043	−0.270 (−0.412)
*p*-value	0.921 (0.033)	0.797	0.111 (0.021)
**Total-IBS-SSS**			
R	0.018	0.063	−0.299 (−0.466)
*p*-value	0.919	0.718	0.091 (0.012)

N = 38. Correlation analyses between heart rate acceleration during tilt test and continuous gastrointestinal symptoms. In brackets significant r- and *p*-value when re-analyzing the group without the patients with organic gastrointestinal disorder (*n* = 6). HR, heart rate; IBS-SSS, Irritable Bowel Syndrom-Severity Scoring System.

### 3.5 Autoantibodies and immunological factors

Microarray for systemic autoimmunity were performed in 34 patients with POTS and in 39 controls, all randomly selected. The most prevalent autoantibodies were Ro-60 (*n* = 6) and Scl-70 (*n* = 4). Ro-60 was equally distributed between POTS and controls, whereas Scl-70 was only found in the POTS cohort. Otherwise, the prevalence of subjects positive for any autoantibody was equal between groups (29.4% in POTS vs 33.3% in controls, *p* = 0.803). No statistically significant differences were found between patients or controls with or without expression of autoantibodies regarding the severity of GI symptoms or tilt test (data not shown).

Baseline serum total tryptase levels were measured in 41 of the 43 patients with POTS. One patient had a slightly elevated tryptase level (12.4 μg/L), according to the upper limit for normal value (11.3 μg/L) (Lab Medicine, 2023b). Another three patients had tryptase levels >8.0 μg/L, which is suggested as the lower level for the most common genetic trait, hereditary *α*-tryptasemia, causing elevated tryptase and symptoms of mast cell activation (Valent et al., 2023). Serum total IgE levels were measured in 40 patients with POTS. Five patients had total IgE levels above the upper limit for normal value (129 kU/L) (Lab Medicine, 2023a). One of these patients reported asthma, whereas the other four patients neither reported any atopic disease, nor any anti-allergy drugs. Total IgE was normal in all four patients with tryptase levels >8.0 μg/L.

## 4 Discussion

The main finding of the present study was that patients with POTS have a high burden of GI- and extraintestinal symptoms, compared with healthy controls. The most prevalent symptoms were nausea, abdominal fullness, and bloating. The most severe GI symptoms were bloating and flatulence, and constipation. GI symptoms were associated with low psychological wellbeing. Importantly, the magnitude of HR acceleration on the initial tilt test was inversely correlated with GI symptoms, i.e., a pronounced HR acceleration at diagnosis was associated with less severe chronic GI symptoms. Finally, there was no overexpression of circulating autoantibodies commonly seen in systemic autoimmune rheumatic diseases, or elevated baseline tryptase levels among POTS patients, and there was no association between autoantibodies and GI symptoms.

To our knowledge, this is the first study where specific GI symptoms have been studied in a POTS cohort in terms of prevalence and the degree of severity on validated GI symptom scales. Additionally, this is the first study that has investigated how the hemodynamic response, as well as signs of autoimmunity, correlated with specific GI- and extraintestinal symptoms.

The results are in line with previous studies examining GI symptoms in POTS (Huang et al., 2013; Wang et al., 2015; Mehr et al., 2018; Tai et al., 2020). In a review by Mehr et al. (2018), pooled data from six original articles showed that the most common, non-cardiovascular symptoms in POTS were nausea and abdominal pain. Conceptually, there is an important difference between orthostatic and non-orthostatic GI symptoms in POTS (Chelimsky and Chelimsky, 2018). Orthostatic GI symptoms, such as nausea and vomiting, can be relieved by recumbent position and cardiovascular treatment strategies (Sullivan et al., 2005; Fortunato et al., 2014), whereas non-orthostatic GI symptoms require other management strategies (Chelimsky and Chelimsky, 2018). Multisystem comorbid conditions are common in POTS, including functional GI disorders (FGID) (DiBaise et al., 2018). However, there is no evidence that POTS itself contribute mechanistically to these conditions, according to a previous study in children (Chelimsky et al., 2015). In the present study, the most severe GI symptoms were bloating and flatulence and constipation. These items are probably non-orthostatic, rather representing comorbidities. Twenty-eight percent of the POTS group reported a diagnosis of IBS, and the median total IBS-SSS score in POTS was 213, corresponding to moderate IBS (Francis et al., 1997). Moreover, 28% reported HSD/EDS and previous research has shown that HSD/EDS is associated with FGIDs, with and without POTS (Beckers et al., 2017; Fikree et al., 2017; Tai et al., 2020). In addition, seven patients reported a concomitant organic GI disorder. Hence, comorbid functional and organic GI disorders may explain the magnitude of GI symptoms in POTS in this study.

Almost all GI symptoms correlated with low psychological wellbeing in the present study. It is well known that low psychological wellbeing is associated with IBS and other FGIDs, where bilateral communication between the brain and the gut results in visceral hypersensitivity (Koloski et al., 2012; Ford et al., 2020). In POTS, previous studies have shown mild to moderate depression and anxiety levels, as well as positive associations between the degree of depression and self-rated orthostatic symptom burden (Raj et al., 2018). Somatic hypervigilance has been described in POTS (Khurana, 2006; DiBaise et al., 2018), suggesting central visceral sensitization (DiBaise et al., 2018). Previous studies regarding FGIDs have shown that lower psychological wellbeing is associated with less effective central pain modulation (Marcuzzi et al., 2019), including an increased membrane excitability, a facilitated synaptic strength, and decreased inhibitory influence on the dorsal horn neurons, all contributing to an abnormal responsiveness to noxious and non-noxious stimuli (Latremoliere and Woolf, 2009; Ossipov et al., 2014). According to a previous study, IBS patients with lowered pain thresholds upon testing also have higher circulating levels of cortisol and corticotrophin, as well as higher blood pressure, indicating a disturbed hypothalamic-pituitary-adrenal axis, possibly due to higher stress levels (Chang et al., 2009; Zhou et al., 2010). It is tempting to believe that these mechanisms contribute to central sensitization and hypervigilance in POTS as well. However, in the setting of the present study, we cannot draw any conclusions whether GI symptoms in POTS are caused by lowered psychological wellbeing with increased hypersensitivity, or *vice versa*.

To further explore the nature of GI symptoms in POTS, correlation tests were performed between symptoms and hemodynamic parameters. Surprisingly, there was a significant inverse correlation between maximum orthostatic HR acceleration at diagnostic tilt test and abdominal pain. The inverse correlation between HR acceleration and various chronic GI symptoms became even more distinct when the patients with organic GI disorders were excluded from the analyses. The same tendency between hemodynamic data and GI symptoms was seen when analyzing the active standing test performed a few years after the tilt test, closer to the recruitment period of the present study.

There are some possible theoretical explanations to the findings regarding the inverse correlation between orthostatic HR acceleration and GI symptoms among POTS patients. First, there may be a possible treatment bias. Patients who present with a pronounced HR acceleration may receive more aggressive treatment or more active follow-up. Active advice on higher salt- and fluid intake, as well as prescription of some cardiovascular drugs, often result in improvement of the typical orthostatic GI symptoms (Sullivan et al., 2005; Fortunato et al., 2014). A second explanation to the finding is that POTS represents a heterogenous syndrome rather than a disease, where multiple mechanisms can affect the phenotypic outcome (Mehr et al., 2018; Fedorowski, 2019). Some of the most important pathophysiological mechanisms behind POTS are (I) a hyperadrenergic state, (II) partial autonomic neuropathy, (III) a persistent predilection for hypovolemia leading to reflex orthostatic tachycardia, and (IV) physical deconditioning (Benarroch, 2012; Tu et al., 2020; Raj et al., 2022). These mechanisms interact; thus, one patient can exhibit characteristics from two or more of these pathophysiological mechanisms (Tu et al., 2020). Signs of autonomic neuropathy and distal peripheral neuropathy are seen in approximately 50% of the POTS population (Low et al., 2009). GI motility disorders are well described in POTS (Huang et al., 2013; Park et al., 2013; Loavenbruck et al., 2015), where up to 18% have delayed gastric emptying, and up to 48% have rapid gastric emptying (Loavenbruck et al., 2015). These disturbances may be considered manifestations of neuropathy. In a study by Gibbons et al. (2013), the HR increasement at tilt test was less pronounced in POTS patients with signs of neuropathy, compared with non-neuropathic POTS. More exaggerated GI symptoms have been reported in neuropathic POTS (Al-Shekhlee et al., 2005). Thus, neuropathic POTS may explain why a subset of patients in the present study exhibit more GI symptoms, but less pronounced HR acceleration. Lastly, more pronounced GI symptoms and less exaggerated orthostatic pulse acceleration were measured in the 28% with self-reported HSD/EDS in the present study. The mechanistic association between POTS and HSD/EDS is not entirely clear (Tai et al., 2020), but common mechanisms behind HSD/EDS and POTS may partly explain the inverse correlations between GI symptoms and HR acceleration in the present study.

Autoantibody microarray assay did not show any difference in expression of autoantibodies commonly found in systemic autoimmune rheumatic disorders, when comparing POTS with controls. Previous studies have shown that comorbid autoimmune disorders in POTS are prevalent (Vernino and Stiles, 2018; Vernino et al., 2021), as in our study, where approximately one-third of participants self-reported such disorders. However, contrasting our study, Blitshteyn (2015) observed in a retrospective study that the prevalence of antinuclear antibodies (ANA) and anti-phospholipid antibodies (aPL) were higher in POTS compared with the normal population. The results were compared with known cut-off values for normal population, and not with comparable controls (Blitshteyn, 2015). Further, others reported ganglionic acetylcholine receptor (AChR) autoantibodies and G-protein coupled receptor autoantibodies being more expressed in POTS, but the pathological significance remains uncertain (Vernino and Stiles, 2018). In the present study, no association was seen between expression of autoantibodies and any GI symptoms, suggesting that systemic autoimmunity is not a major driver of GI symptoms in POTS.

Allergic comorbidity and medications were common in the present study, and total IgE was elevated in a subset of patients. However, baseline total serum tryptase was only slightly elevated in one patient. GI symptoms were not more pronounced in patients with self-reported allergic disorders or on anti-allergic medications. Thus, the present study could not confirm the results by Kohno et al. (2021), who were suggesting that mast cell activation is a major driver of atypical POTS symptoms such as GI complaints.

The strength of this study is use of different validated scoring systems for assessment of GI symptom burden. The patients were thoroughly examined at a tertiary cardiovascular center with a syncope unit. The tilt data was present in all patients, and active standing test was performed in 88% of the patients at a later time point, which had the same correlation tendency to GI symptoms as the tilt test did, reflecting robustness of the findings. In addition, all patients were examined clinically at inclusion.

One limitation to the study may be a selection bias since POTS patients were recruited from a highly specialized center, and thus, might have more exaggerated symptoms than the POTS population in general. Furthermore, 82 patients with POTS were invited to the study, but only 43 ended up at inclusion. This is a limitation that has some explanations. Firstly, we wanted to examine the patients physically, and many patients declined to participate in the study due to long distance to the study center. In addition, some of the patients were hindered by severe orthostatic symptoms. Lastly, the study was conducted during the covid pandemic, and some patients were excluded due to covid symptoms. Another limitation was that a control group recruited from hospital staff might be healthier with less symptoms than controls recruited more randomly. All data on comorbidities were self-reported, which is especially problematic when it comes to HSD/EDS, as these diagnostic terms are used inconsistently among patients according to previous studies (Tai et al., 2020). Both HSD and EDS present with musculoskeletal symptoms and joint hypermobility, but whereas EDS is a rare disorder of the connective tissue with various systemic manifestations, HSD is more common, affecting approximately 3% of the population, but without systemic manifestations (Miller et al., 2020). However, the prevalence rates regarding self-reported HSD/EDS were fairly in line with previous studies (Miller et al., 2020; Tai et al., 2020). The patients were not asked whether GI symptoms worsened upon standing, which would have been interesting, to discriminate between orthostatic and non-orthostatic GI symptoms. Another limitation was that the tilt tests were performed some years ago in several patients, although the diagnosis was confirmed by active standing test during enrolment. Regarding measurement of baseline tryptase, the study was not designed to diagnose mast cell activation syndrome, where tryptase must be measured both at baseline, and upon acute symptoms. The patients were not fasting at blood sampling, and two patients were on oral mast cell stabilizing therapy. Finally, pharmacological treatment was abundant among POTS patients. Several of the reported agents, including opioids and cholinesterase inhibitors, are known to cause adverse GI symptoms (Kanjwal et al., 2011; Drewes et al., 2016).

A recent Indian study (Inbaraj et al., 2023) has investigated heart rate variability (HRV) in POTS, based on the assumption that HRV is the most reliable marker of cardiovascular autonomic dysfunction, showing affected HRV parameters in POTS compared with controls (Inbaraj et al., 2023). Based on the results from that study, future studies would be to further investigate the relationship between GI symptoms and parameters of HRV in POTS.

## 5 Conclusion

Gastrointestinal symptoms are common in POTS and are associated with low psychological wellbeing. More pronounced tachycardia during tilt testing or active standing test is associated with less severe GI complaints in POTS. This finding may reflect different contributing pathophysiological mechanisms and comorbidities in POTS. Finally, this study did not find any evidence supporting that POTS is associated with conventional biomarkers of autoimmunity.

## Data Availability

The original contributions presented in the study are included in the article/Supplementary Material, further inquiries can be directed to the corresponding author.
