# Supplementary material for: Gastrointestinal symptoms in patients with postural orthostatic tachycardia syndrome in relation to hemodynamic findings and immunological factors
Source: Front Physiol. 2024 Jan 29;15:1342351. doi: 10.3389/fphys.2024.1342351 (PMC10859499; doi:10.3389/fphys.2024.1342351)
Supplement: Supplementary file 1 [file DataSheet1.pdf]

Supplementary Material

Supplementary Table 1.

**Antigen**	**Protein**	**Peptid sequence**
Cit Fibα$_{36\text{-}50}$	Fibrinogen α-chain, 36-50	GP(cit) VVE (cit)HQ SAC KDSK
Cit Fibβ$_{60\text{-}74}$	Fibrinogen β-chain, 60-74	(cit)PA PPP ISG GGY (cit)A(cit)
Cit Fibβ$_{36\text{-}52}$	Fibrinogen β-chain, 36-52	NEE GFF SA(cit) GHR PLD KK
Cit Fil$_{307\text{-}324}$	Filaggrin, 307-324	HQC HQE ST(cit) GRS RGR CGR SGS
Acet Fil$_{307\text{-}324}$	Filaggrin, 307-324	HQC HQE ST (AC) GRS RGR CGR SGS
Carb Fil$_{307\text{-}324}$	Filaggrin, 307-324	HQC HQE ST (HC) GRS RGR CGR SGS
Cit H3$_{1\text{-}30}$	Histone 3, 1-30	A(cit)T KQT A(cit)K STG GKA P(cit)K QLA TKA A(cit)K SAP
Cit H3$_{21\text{-}44}$	Histone 3, 21-44	ATK AA(cit)KSA PAT GGV KKP H(cit)Y (cit)PG GGK
H4$_{1\text{-}18}$	Histone 4, 1-18	SGR GKG GKG LGK GGA KRH RKVL
Acet H4$_{1\text{-}18\ \text{-Ac8, 12,16}}$	Histone 4, 1-18	SGR GKG GK(Ac)G LGK(Ac) GGA K(Ac)RH RKVL
Acet H4$_{1\text{-}18\ \text{-Ac16}}$	Histone 4, 1-18	SGR GKG GKG LGK GGA K(Ac)RH RKVL
Acet H4$_{1\text{-}18\ \text{-Ac5}}$	Histone 4, 1-18	SGR GK(Ac)G GKG LGK GGA KRH RKVL
Cit TNC1	Tenascin, 2026-2040	VFL RRK NG(cit) ENF YQNW
Cit TNC5	Tenascin, 2176-2200	EHS IQF AEM KL(cit) PSN F(cit)N LEG (cit)(cit)KR
Cit Vim$_{60\text{-}75}$	Vimentin, 60-75	VYA T(cit)S SAV (cit)L(cit) SSVP
CENP B	Centromer protein B	
dsDNA	Double stranded DNA	
Fibrillarin	Fibrillarin	
Jo-1	Histidyle-tRNA synthetase	
Mi-2	Mi-2 nuclear antigen	
PCNA	Proliferating Cell Nuclear Antigen	
PM-Scl100	Also known as Rrp6	
Rip P0	Phosphorylated P0 protein of the ribosomalstalk	
Rip P1	Phosphorylated P1 protein of the ribosomalstalk	
Rip P2	Phosphorylated P2 protein of the ribosomalstalk	
RNA Pol III	RNA Pol III	
RNP70	U1-snRNP complex, 70kD protein	
RNP A	U1-snRNP complex, protein A	
RNP C	U1-snRNP complex, protein C	
Ro52	Also known as TRIM21	

Ro60	Also known as TROVE2	
Scl-70	70 kD immunoreactive fragment of Topoisomeras I	
Sm	Smith protein complex	
SmB/B'	Smith protein complex B protein	
SS-B/La	Sjögren syndrome type B antigen/ Lupus La antigen	

Protein sequences not shown. Some peptid sequences not shown due to undisclosed information by Thermo Fisher Scientific Immunodiagnostics, Uppsala, Sweden.

Supplementary Table 2. Comorbidities

**Comorbidities**		**POTS (N=43)**	**Healthy controls (N=74)**
**Gastrointestinal disorders**			
	Irritable bowel syndrome	12	
	Obstipation	3	
	Gastroparesis	3	
	Celiac disease	2	
	Inflammatory bowel disease	2	
	Microscopic colitis	2	
	Dyspepsia	2	1
	Polyposis syndrome	1	
	Lactose intolerance	1	
	Gastroesofageal reflux disorder/Peptic ulcer disease	1	
	Diverticulitis	1	
	Cholelithiasis	1	
**Rheumathological and musculosceletal disorders**			
	HSD/EDS	12	
	Fibromyalgia	2	

	Lumbago/herniated disc/scoliosis	2	
	Psoriasis arthritis	1	
	Raynaud syndrom	1	
	Whiplash injury	1	
	Back injury	1	
	Meniscus injury	1	
**Psychiatric diseases**			
	Neuropsychiatric disorders (ADHD/ADD/Autism spectrum disorders)	6	
	Depression and anxiety disorders	4	
	Eating disorders	1	
	Bipolar disorder	1	
**Asthma and allergy**			
	Asthma	9	1
	Allergy	2	4
**Gynaecological disorders**			
	Endometriosis	5	
	Myoma	1	
	Polycysic ovarian syndrome	1	
	Premenstrual dysphoric disorder		1
**Neurological disorders**			
	Migraine	7	1
	Tinnitus	1	1
**Endocrine disorders**			
	Thyroid disease (hypothyroidism, goiter, graves thyrotoxicosis)	6	
	Diabetes Mellitus II	1	
**Cardiovascular diseases**			
	Sick sinus syndrome	2	
	Inappropriate sinus tachycardia	2	
	Pulmonary embolism	1	
**Skin diseases**			

	Rosacea	1	1
	Lichen sclerosus	1	
	Eczema		2
	Psoriasis		1
**Disorders of the urinary tract**			
	Renal disease	1	
	Urinary bladder dysfunction	1	
**Miscellaneous**			
	Myalgic encephalomyelits	5	
	Mast cell activation	4	
	Post-Covid	3	
	Exhaustion disorder	1	

ADHD=Attention-Deficit/Hyperactivity Disorder, ADD=Attention-Deficit Disorder, HSD/EDS=hypermobile spectrum disorder/Ehlers-Danlos syndrome

Supplementary Table 3. Pharmacological medications

		**POTS N=43**	**Healthy controls N=61**
**Cardiovascular and POTS-related drugs**			
	Antihypotensive agents	17	0
	Ivabradine I(f) receptor inhibitors	16	0
	Beta blockers	13	0
	Central stimulating agents	8	0
	Cholinesterase inhibitors	6	0
	Mineral corticoids	3	0
	Short acting nitroglycerine	3	0
	Calcium channel blockers	2	0
	Angiotensin receptor blockers	1	0
**Asthma and allergy**			
	Histamine H1-blockers	13	2
	Inhaled β2-agonists	7	0
	Leukotriene receptor agonists	5	0
	Inhaled steroids	4	0
	Sodium Cromoglicate	3	0

	Nasal steroids	2	1
	Inhaled ipratropiumbromide	1	0
	Oral budesonide	1	0
	Eye drops	0	1
	Topical steroids	0	1
**Hormonal drugs**			
	Combined hormonal contraceptives	8	3
	Progesterone	6	1
	Thyroid hormones	5	0
	GLP-1-analogs	1	0
**Pain killers**			
	Acetaminophen	6	1
	Opioids and opioid-like drugs	5	0
	Non-steroidal Anti-Inflammatory Drugs	3	1
	Muscle relaxing agents	2	0
	Gabapentinoids	2	0
	Spasmolytics	1	0
**Vitamin and mineral supplementation**			
	B12 (oral or injected)	6	0
	D-vitamins	4	0
	Multivitamins	3	2
	Folic acid	2	0
	Potassium chloride	1	0
	Magnesium	1	0
	Sodium chloride tablets	1	0
**Antidepressants and mood stabilizers**			
	Selective serotonin reuptake inhibitor	7	2 (of which one cyclic due to PMDD)
	Serotonin and noradrenaline reuptake inhibitors	3	0
	Noradrenaline reuptake inhibitors	3	0
	Antiepileptic drugs	2	0
	Tricyclic antidepressants	2	0
	Other antidepressants	1	0
**Gastrointestinal drugs**			
	Histamine H2-blockers	7	0
	Proton pump inhibitors	2	1
	5HT3-antagonists	2	0
	5HT4-antagonists	2	0
	Laxatives	1	0

	Bulking agents	0	1
**Sleeping pills**			
	Melatonin	4	1
	Benzodiazepine-like	2	1
	Others	3	0
**Tranquilizers**			
	Non-benzodiazepines	3	0
	Benzodiazepines	1	0
**Miscellaneous**			
	Triptans	2	0
	Statins	2	0
	TNF-α antagonist	1	0
	Direct oral anticoagulants	1	0

GLP=glucagon like peptide. PMDD=premenstrual dysphoric disorder. TNF=tumor necrosis factor.

Supplementary 4. GI symptoms in POTS patients with and without organic GI disorder

	Organic disorder (N=7)	No organic disorder (n=36)	p-value
**Gastroparesis score**			
Loss of appetite	5 (71.4%)	15 (41.7%)	0.222
Dysphagia	3 (42.9%)	13 (36.1%)	1.000
Meal-related cough	3 (42.9%)	11 (30.6%)	0.665
Early satiety	2 (28.6%)	21 (58.3%)	0.222
Nausea	6 (85.7%)	28 /77.8%)	1.000
Vomiting	2 (28.6%)	4 (11.4%) 1 missing	0.257
Weight loss	3 (42.9%)	10 (28.6%) 1 missing	0.657
Abdominal fullness	5 (71.4%)	25 (69.4%)	1.000
Bloating	6 (100 %), 1 missing	22 (61.1%)	0.083
Regurgitation	4 (57.1%)	19 (52.8%)	1.000
Constipation	3 (42.9%)	25 (69.4%)	0.215
Diarrhea with gas	4 (57.1%)	19 (52.8%)	1.000
Evacuation incontinence	3 (42.9%)	7 (19.4%)	0.325
Postprandial perspiration	4 (57.1%)	13 (36.1%)	0.407
**VAS-IBS**			
Abdominal pain	31 (30–52)	30 (16–63)	0.818
Diarrhea	60 (0–69)	18 (0–64)	0.464
Constipation	15 (0–73)	63 (12–75)	0.356
Bloating and flatulence	80 (62–96)	64 (18–83)	0.199
Vomiting and nausea	57 (10–80)	42 (23–70)	0.921

Psychological well-being	60 (22–82)	50 (28–60)	0.270
Symptoms' influence on daily life	76 (74–100)	49 (21–73)	0.054
Urgency	2 (28.5%)	15 (42.9%) 1 missing	0.681
Incomplete evacuation	5 (71.4%)	25 (69.4%)	1.000
**IBS-SSS**			
Total IBS-SSS	320 (198–392)	188 (129–300) 3 missing	0.140
Nausea	57 (10–80)	42 (23–70)	0.921
Early satiety	51 (5–64)	28 (3–59)	0.541
Headache	75 (23–90)	71 (38–85)	0.805
Back pain	50 (43–68)	50 (11–82)	0.974
Lethargy	90 (58–98)	92 (82–100)	0.678
Excess wind	50 (38–82)	61 (28–83)	0.921
Heart burn	19 (3–71)	25 (4–60)	0.644
Urinary symptoms	7 (0–76)	54 (19–85)	0.217
Thigh pain	45 (2–60)	40 (2–57)	0.960
Bodily pain	76 (67–80)	73 (24–98)	0.908
Total extraintestinal score	285 (181–354)	272 (160–333)	0.921

Values presented as median (interquartile range), or number (percent). Comparative analyses were performed with Mann Whitney-U test or Fisher's Exact test. VAS-IBS= Visual Analog Scale for Irritable Bowel Syndrome. IBS-SSS= Irritable Bowel Syndrom-Severity Scoring System.

Supplementary table 5. GI symptoms in POTS patients with and without asthma/allergy/anti-allergic drugs

	Allergy (N=15)	No allergy (n=28)	p-value
**Gastroparesis score**			
Loss of appetite	8 (53.3%)	12 (42.9%)	0.540
Dysphagia	7 (46.7%)	9 (32.1%)	0.509
Meal-related cough	8 (53.3%)	6 (21.4%)	0.046
Early satiety	9 (60.0%)	14 (50.0%)	0.749
Nausea	12 (80.0%)	22 (78.6%)	1.000
Vomiting	3 (20.0%)	3 (11.1%) 1 missing	0.649
Weight loss	3 (21.4%) 1 missing	10 (35.7%)	0.485
Abdominal fullness	8 (53.3%)	22 (78.6%)	0.162
Bloating	10 (66.7 %)	18 (66.7%) 1 missing	1.000
Regurgitation	8 (53.3%)	15 (53.6%)	1.000
Constipation	10 (66.7 %)	18 (64.3%)	1.000
Diarrhea with gas	10 (66.7 %)	13 (46.4%)	0.336

Evacuation incontinence	3 (20.0%)	7 (25.0%)	1.000
Postprandial perspiration	6 (40.0%)	8 (28.6%)	0.507
**VAS-IBS**			
Abdominal pain	30 (19–61)	31 (12–63)	0.574
Diarrhea	60 (0–77)	18 (0–54)	0.241
Constipation	24 (11–73)	63 (8–77)	0.475
Bloating and flatulence	65 (50–90)	68 (18–82)	0.674
Vomiting and nausea	54 (31–70)	33 (13–72)	0.189
Psychological well-being	50 (32–60)	50 (28–64)	0.980
Symptoms' influence on daily life	62 (20–85)	54 (24–75)	0.557
Urgency	6 (42.9%) 1 missing	11 (39.3%)	1.000
Incomplete evacuation	10 (66.7%)	20 (71.4%)	0.742
**IBS-SSS**			
Total IBS-SSS	264 (131–335) 1 missing	201 (128–310) 2 missing	0.571
Nausea	54 (31–70)	33 (13–72)	0.189
Early satiety	39 (5–85)	35 (4–59)	0.818
Headache	81 (67–90)	61 (26–80)	0.054
Back pain	58 (23–71)	48 (8–82)	0.702
Lethargy	94 (85–100)	91 (80–98)	0.433
Excess wind	70 (22–83)	50 (29–82)	0.646
Heart burn	22 (2–70)	24 (9–57)	0.838
Urinary symptoms	83 (19–89)	37 (8–73)	0.068
Thigh pain	51 (4–60)	15 (2–50)	0.210
Bodily pain	78 (66–90)	71 (17–98)	0.507
Total extraintestinal score	315 (205–356)	259 (153–316)	0.177

Values presented as median (interquartile range), or number (percent). Comparative analyses were performed with Mann Whitney-U test or Fisher's Exact test. VAS-IBS= Visual Analog Scale for Irritable Bowel Syndrome. IBS-SSS= Irritable Bowel Syndrom-Severity Scoring System.
